# Improving the Nutritional Status of Adolescent Females in Gujarat: The Case for Targeted Investment

**DOI:** 10.7759/cureus.29731

**Published:** 2022-09-29

**Authors:** Hardik Parmar, Mrunal Mehta, Manoj S Patil, Somen Saha, Deepak Saxena

**Affiliations:** 1 Public Health, Indian Institute of Public Health, Gandhinagar, IND; 2 Research and Development, Jawaharlal Nehru Medical College, Datta Meghe Institute of Medical Sciences, Wardha, IND; 3 Public Health, Jawaharlal Nehru Medical College, Datta Meghe Institute of Medical Sciences, Wardha, IND

**Keywords:** malnutrition, thinness, anemia, overweight, undernutrition

## Abstract

Introduction

Undernutrition is one of the key determinants of morbidity and mortality in adolescent females worldwide and in India. Malnutrition, particularly undernutrition, is highly prevalent among adolescent females. Although undernutrition affects the health status of adolescent females leading to poor growth and developmental problem issues among them, still, the adolescent group remains to be neglected group. The present paper particularly focuses on challenges and ways forward for improving the nutritional status of adolescent females in Gujarat.

Methods and material

It’s a mixed method study where the secondary data analysis was conducted comparing the National Family Health Survey-5 (NFHS-5) report with the Comprehensive National Nutritional Survey (CNNS) report for the assessment of undernutrition, overweight, and anemia status among adolescent females, and the primary assessment of the nutritional status of adolescent females across Gujarat was conducted through anthropometric measurements of height and weight.

Results

The NFHS-5 report findings showed total thinness among adolescent females (15-19 years) in Gujarat to be 52.5%, which increased by 3% from the NFHS-4 findings. Anemia among adolescent females has also been reported to be 69%, which also increased by 12.5% from the NFHS-4 findings. Tribal regions/populations had a higher prevalence of undernutrition. Being overweight among urban adolescent females was more prevalent than in rural regions. The key findings of the CNNS report also showed that 24% of adolescent females (10-19 years) were thin while 5% of adolescent females were overweight/obese in India, while in Gujarat, 8% of adolescent females were overweight/obese. The primary data gathered suggest a prevalence of overweight in Gujarat of 8.9% in adolescent females and total thinness of 50%.

Conclusion

The nutritional status of adolescent females is still a major concern in many parts of India. Considering the complex set of challenges to tackle malnutrition in Gujarat and with specific attention to the adolescent group, it is vital to understand district-specific challenges and plan, program, and design district-specific strategies and implement actions to improve the existing nutritional status of adolescent females.

## Introduction

Adolescents constitute over 20% of India’s population. The World Health Organization (WHO) has identified adolescence as “the period of the life span of the ages between 10 and 19 years” [1]. Adolescent females suffer from various forms of malnutrition because of their increased nutritional needs and low social power [2]. Nutritional deficiencies have widespread consequences, especially in adolescent females. A malnourished female affects her overall health, well-being, and productivity. Further, a malnourished female is likely to give birth to undernourished children, which transcends undernutrition toward future generations. Adolescence is also identified as the second window of opportunity after the first thousand days of life to improve nutritional inadequacies and inadequate growth and development from childhood [3]. Unfortunately, adolescents have been a neglected group. Very little information is available on the influence of their social, economic, cultural, health, and food environments in relation to health and nutritional status [4-7].

Malnutrition, particularly undernutrition, is in greater prevalence among adolescents in low- and middle-income countries [8]. Nutritional status among adolescents is a key determinant of health outcomes; undernutrition affects the health status of adolescent females. In addition to causing mortality, it also causes long-lasting effects on the growth, development, and physical fitness of survivors [9-12]. Malnourished children and adolescents are also at higher risk for impaired growth, low immunity, poor cognitive development, and mortality [13].

Several programs are being implemented in India on improving adolescent nutritional status. These include the Prevention of Under-Nutrition and Reduction of Nutritional Anaemia (PURNA) scheme targeting the prevention of undernutrition and the reduction of nutritional anemia among adolescent females with PURNA shakti packets, iron-folic acid (IFA) pills, and health and nutrition education services for both school-going and non-school-going adolescent females of 15-18 years. The Rashtriya Kishor Swasthya Karyakram or RKSK program aimed to improve nutrition by reducing the prevalence of malnutrition among adolescent females and males along with organizing adolescent health days to mobilize adolescent age-groups through information, education, and communication activities. RKSK also emphasized on strengthening Adolescent Friendly Health Clinics (AFHC) under the umbrella of facility-based interventions and additional weekly supplementation of IFA under the Weekly Iron-Folic Acid Supplementation (WIFS) program for school-going adolescents.

Poor nutrition among adolescents in Gujarat is a serious concern despite being a progressive and economically well-off state. Gujarat is among one of the priority states in the area of nutrition. Among various other challenges, one of the underlying challenges being the lack of effective implementation of programs prevails, which led to the poor nutritional status of adolescent females in Gujarat. This paper particularly focuses on the nutritional status of adolescent females in Gujarat.

## Materials and methods

Unit-level data from the National Family Health Survey-5 (NFHS-5) were analyzed for a detailed review on the nutritional status of adolescent females aged 15-19 years (N=3,762 females from Gujarat). The Comprehensive National Nutritional Survey (CNNS) 2016-2018 assessed undernutrition and overweight status and food consumption among adolescents. The CNNS report includes adolescents in 10-19-year-old age-group. In addition, a primary survey on 8,679 adolescent females aged 11-18 years in Gujarat in 2021 led to an in-depth understanding of the determinants of malnutrition. The primary assessment of the nutritional status of adolescent females across all districts and major corporations of Gujarat state was conducted through anthropometric measurements of height and weight. The surveys assessed body mass index (BMI), measured as kg/m^2^ to assess thinness, overweight, and obesity. As per WHO classification, BMI of less than or equal to 17 kg/m^2^ was classified as moderately/severely thin, between 17 and 18.5 as thin, between 18.5 and 25 as normal, and more than 25 as overweight/obese.

The comparative analysis of the nutritional status of adolescent females was conducted between the data sources of the National Family and Health Survey (NFHS), Comprehensive National Nutritional Survey (CNNS), and primary assessment (field survey) to understand the level of nutritional deficiencies among adolescent females in Gujarat. Further, primary assessment findings for all districts and major corporations of Gujarat were used to classify districts and corporations in order of priority based on the nutritional status of adolescent females for severe and moderate thinness and overweight category. High-priority districts are defined as those districts performing worse than the state’s average in the areas of severe and moderate thinness. The study was approved by the Institutional Ethics Committee of the Indian Institute of Public Health, Gandhinagar (approval number 04/2021-22, dated 07/06/2021).

## Results

Nutritional status of adolescent females

Out of 3,762 females between 15 and 19 years assessed in NFHS-5, 52.5% are totally thin (BMI: <18.5), and 27.4% of adolescent females (15-19 years) are moderately/severely thin (Table 1). CNNS data report that 40.7% are totally thin while 10.5% were moderately or severely thin in the age-group of 10-19 years. Our primary survey nearly mirrored the data of NFHS-5. Overweight/obesity ranged from 4.9% to 8.9% across the surveys.

Roughly, two in every three adolescents were anemic. CNNS survey shows that 22% of adolescents had iron deficiency (low serum ferritin) and female adolescents had a higher prevalence of iron deficiency (31%) than male adolescents (12%). Adolescents in urban areas had a higher prevalence of iron deficiency compared to their rural counterparts.

**Table 1:** Nutritional status: adolescent females in Gujarat Note: Measures were derived for aged 15-19 years based on NFHS-5 unit-level data. CNNS refers to the age-group 10-19 years; primary survey refers to aged 11-19 years NFHS-5: National Family Health Survey-5; CNNS: Comprehensive National Nutritional Survey; BMI: body mass index; NA: not available

Adolescent females	NFHS-5 (2019-2020)	CNNS (2016-2018)	Primary survey (2021)
Moderately/severely thin (BMI: ≤17.0)	27.40%	10.50%	22.16%
Thin (BMI: >17.0-<18.5)	25.10%	30.20%	28.23%
Normal (BMI: ≥18.5-<25.0)	42.60%	51.40%	40.71%
Overweight/obese (BMI: ≥25.0)	4.90%	8.00%	8.91%
Mild anemia (11-11.9 g/dL)	25.90%	17.80%	NA
Moderate anemia (8-10.9 g/dL)	39.40%	12.90%	NA
Severe anemia (<8 g/dL)	3.70%	2.70%	NA

High-priority districts of Gujarat

**Figure 1:**
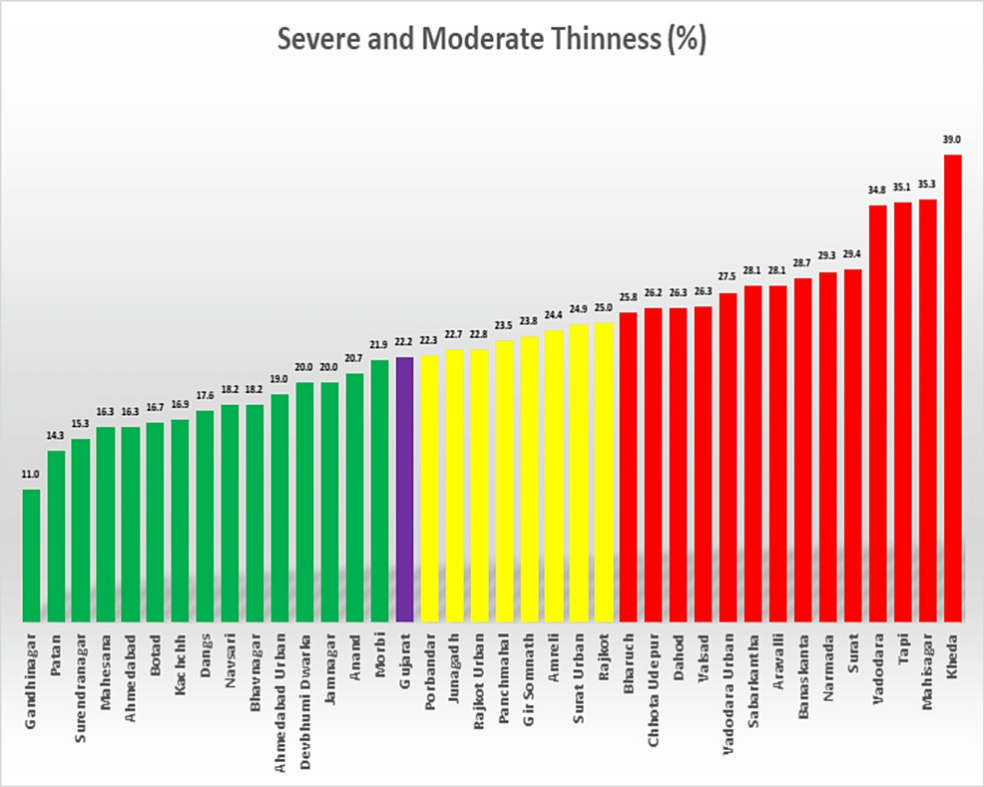
Percentage of severe and moderate thinness among adolescent females aged 11-18 years by districts, Gujarat

As Figure 1 shows, 22 districts of Gujarat score above the state average on severe and moderate thinness and can be classified as high-priority districts. These are primarily the tribal-dominated districts of the state.

## Discussion

The nutritional status of adolescent females is often not emphasized adequately as programs and schemes largely catered toward pregnant-lactating mothers and children [14]. In addition, there are several structural and functional challenges in the implementation of existing schemes for adolescents. Resource constraints of programmatic division, skill gaps regarding counseling, and technical skills are some of the major challenges. The current review from two different databases and a primary survey specifically highlights the critical burden of malnutrition among adolescent females in Gujarat. It further highlights the districts that need urgent attention. Geographical acceptance and availability of limited food groups in the dietary regimes are some of the factors in tribal regions of Gujarat associated with the high prevalence of thinness among adolescent females in Gujarat. The limited evidence suggests that a compromised diet in terms of quantity, quality, and diversity increases micronutrient deficiencies, morbidity, and disease burden, which indicates the presence of dual burden of malnutrition and unhealthy lifestyles and the emergence of early onset of diet-related chronic disorders in the adolescent population [15,16]. A comparative analysis of such dietary regimes across districts would be helpful to prepare a Dietary Diversity Index (DDI) of different districts and corporations of Gujarat, which in turn can help the administration and government to design programs and policies to influence the nutritional status at the community level in the future.

Targeted intervention in high-priority districts is required to advocate program- and policy-level changes on improving the nutritional status of adolescent females rapidly in Gujarat. Being overweight among adolescents is also emerging as a major problem, and strikingly, urban settings are facing this challenge, which requires actions at multiple levels starting from food consumption behavior, physical activities, and healthy eating [17].

Further, the ongoing PURNA scheme should be strengthened by promoting the practices of consuming PURNA shakti packets along with IFA pill for both school-going and non-school-going adolescent females. Community-level health and nutrition education-related programs and meetings are also important components of the PURNA scheme, which needs to be strengthened particularly in poor-performing districts and blocks to improve the nutritional status of adolescent females. There need to be convergence and coordination between health and women and child development (WCD) departments for regular screening of hemoglobin status and nutritional deficiencies through effective engagement of the WCD team and the Rashtriya Bal Swasthya Karyakram (RBSK) team at the village, block, and district levels. It is also imperative that nutritional services provided under the Integrated Child Development Scheme (ICDS) program need to be upgraded by promoting and incorporating locally available and acceptable food groups to improve the nutritional status of adolescent females, along with the advocacy of hot cooked meal for adolescent females.

The nutritional status of adolescent females is a major concern of Gujarat as more than two-thirds of districts and corporations require specific attention to address challenges for improving nutritional status. Dietary diversity and food choices play a major role in shaping the nutritional status of population groups [18]. Therefore, a Dietary Diversity Index (DDI) among adolescent females and exploring locally available food groups are required to improve the nutritional status of adolescent females.

There are some limitations of the study that involve different timeframe of the resource database reviewed. The Comprehensive National Nutrition Survey (CNNS) report was published in 2016, while the National Family Health Survey (NFHS)-5 reports was published in 2019-2020. Further, primary assessments of the nutritional status of adolescent females were conducted during the year 2021. Hence, there is a 3-4-year gap between the secondary database used for critical review, which could affect the data accuracy. Further, CNNS report covers the nutritional status of aged 10-19 years, while the NFHS report covers the nutritional status report of 15-19-year-old adolescent females. Also, primary survey compared the NFHS-5 with the CNNS nutritional status data of 11-18-year-old females.

## Conclusions

Considering the complex set of challenges to tackle malnutrition in Gujarat and with specific attention to the adolescent group, it is vital to understand district-specific challenges and plan, program, and design district-specific strategies and implement actions to improve the existing situations.

## References

[1] WHO WHO (2016). Underweight and overweight in adolescents and young women. Pan Am Health Organ.

[2] Choudhary S, Mishra C, Shukla KP (2003). Nutritional status of adolescent girls in rural area of Varanasi. Indian J Prev Soc Med.

[3] Ghai OP, Gupta P (2000). Essential paediatrics. Essential Paediatrics. 6th edition. CBS Publishers and Distributors.

[4] Patton GC, Sawyer SM, Santelli JS (2016). Our future: a Lancet commission on adolescent health and wellbeing. Lancet.

[5] Mokdad AH, Forouzanfar MH, Daoud F (2016). Global burden of diseases, injuries, and risk factors for young people’s health during 1990-2013: a systematic analysis for the Global Burden of Disease Study 2013. Lancet.

[6] Patton GC, Coffey C, Cappa C (2012). Health of the world’s adolescents: a synthesis of internationally comparable data. Lancet.

[7] Blum RW, Nelson-Mmari K (2004). The health of young people in a global context. J Adolesc Heal.

[8] Ng M, Fleming T, Robinson M (2014). Global, regional, and national prevalence of overweight and obesity in children and adults during 1980-2013: a systematic analysis for the Global Burden of Disease Study 2013. Lancet.

[9] Cappa C, Wardlaw T, Langevin-Falcon C, Diers J (2012). Progress for children: a report card on adolescents. Lancet.

[10] Kulin HE, Bwibo N, Mutie D, Santner SJ (1982). The effect of chronic childhood malnutrition on pubertal growth and development. Am J Clin Nutr.

[11] Blössner M, Onis M (2005). Malnutrition: quantifying the health impact at national and local levels. https://apps.who.int/iris/bitstream/handle/10665/43120/9241591870.pdf.

[12] Saunders J, Smith T (2010). Malnutrition: causes and consequences. Clin Med (Lond).

[13] Black R, Victora C, Walker S (2013). Maternal and child undernutrition and overweight in low-income and middle-income countries. Lancet.

[14] (2022). World Health Organization: Adolescent nutrition: a review of the situation in selected South-East Asian countries. https://apps.who.int/iris/handle/10665/204764.

[15] Twig G, Yaniv G, Levine H (2016). Body-mass index in 2.3 million adolescents and cardiovascular death in adulthood. N Engl J Med.

[16] Akseer N, Al-Gashm S, Mehta S, Mokdad A, Bhutta ZA (2017). Global and regional trends in the nutritional status of young people: a critical and neglected age group. Ann N Y Acad Sci.

[17] Young MF, Nguyen P, Tran LM, Avula R, Menon P (2020). A double edged sword? Improvements in economic conditions over a decade in India led to declines in undernutrition as well as increases in overweight among adolescents and women. J Nutr.

[18] Nithya DJ, Bhavani RV (2018). Dietary diversity and its relationship with nutritional status among adolescents and adults in rural India. J Biosoc Sci.

